# Differential post-transcriptional regulation of IL-10 by TLR2 and TLR4-activated macrophages

**DOI:** 10.1002/eji.201343734

**Published:** 2013-12-16

**Authors:** Maria Teixeira-Coelho, Joana Guedes, Pedro Ferreirinha, Ashleigh Howes, Jorge Pedrosa, Fernando Rodrigues, Wi S Lai, Perry J Blackshear, Anne O'Garra, António G Castro, Margarida Saraiva

**Affiliations:** 1 Life and Health Sciences Research Institute (ICVS), School of Health Sciences, University of MinhoBraga, Portugal; 2 ICVS/3B's – PT Government Associate Laboratory, Braga/GuimarãesPortugal; 3 The MRC National Institute for Medical ResearchLondon, United Kingdom; 4 Laboratory of Signal Transduction, National Institute of Environmental Health SciencesResearch Triangle Park, NC, USA; 5 Instituto de Biologia Molecular e Celular (IBMC)Porto, Portugal; 6 Instituto de Biologia Molecular e Celular (IBMC), Instituto de Ciências Biomédicas de Abel Salazar (ICBAS), University of PortoPorto, Portugal

**Keywords:** IL-10, MAPKs, Post-transcriptional regulation, TLRs

## Abstract

The activation of TLRs by microbial molecules triggers intracellular-signaling cascades and the expression of cytokines such as IL-10. *Il10* expression is tightly controlled to ensure effective immune responses, while preventing pathology. Maximal TLR-induction of *Il10* transcription in macrophages requires signaling through the MAPKs, ERK, and p38. Signals via p38 downstream of TLR4 activation also regulate IL-10 at the post-transcriptional level, but whether this mechanism operates downstream of other TLRs is not clear. We compared the regulation of IL-10 production in TLR2 and TLR4-stimulated BM-derived macrophages and found different stability profiles for the *Il10* mRNA. TLR2 signals promoted a rapid induction and degradation of *Il10* mRNA, whereas TLR4 signals protected *Il10* mRNA from rapid degradation, due to the activation of Toll/IL-1 receptor domain-containing adaptor inducing IFN-β (TRIF) and enhanced p38 signaling. This differential post-transcriptional mechanism contributes to a stronger induction of IL-10 secretion via TLR4. Our study provides a molecular mechanism for the differential IL-10 production by TLR2- or TLR4-stimulated BMMs, showing that p38-induced stability is not common to all TLR-signaling pathways. This mechanism is also observed upon bacterial activation of TLR2 or TLR4 in BMMs, contributing to IL-10 modulation in these cells in an infection setting.

## Introduction

TLRs play an important role in the recognition of microorganisms and initiation of innate immune responses, by recognizing different molecular patterns in microbes 1. TLR triggering, through the activation of the signaling adaptors MyD88 and/or Toll/IL-1 receptor domain containing adaptor inducing IFN-β (TRIF), leads to a specific cellular transcriptional program with the expression of different immune mediators, such as pro-inflammatory cytokines 1. In addition to pro-inflammatory cytokine secretion, TLR signaling also leads to the production of IL-10 by innate immune cells. IL-10 is a powerful anti-inflammatory cytokine produced by many cells of the immune system, including macrophages 2. Strict regulation of the balance between IL-10 production and the pro-inflammatory immune response during infection is essential to achieve clearance of the pathogen in the absence of immunopathology, while at the same time avoiding the establishment of chronic infection 3 IL-10 production by TLR4-activated macrophages or dendritic cells (DCs) requires both MyD88 and TRIF signals 4. The recruitment of both TNF receptor associated factor 6 5 and TNF receptor associated factor 3 6 by either adaptor is fundamental for IL-10 production. Overall, whereas the molecular pathways mediated by MyD88 and implicated in IL-10 regulation are well understood, less is known of the specific contribution of TRIF.

Several mechanisms for *Il10* gene regulation have been described, including epigenetic control, the activation of specific intracellular-signaling cascades, the action of certain transcription factors, and post-transcriptional control 2. Although these broad mechanisms likely operate in all IL-10-producing cells, cell-specific factors have also been described 2. For example, a specific NF-κB-binding enhancer sequence at the *Il10* locus regulates *Il10* transcription in macrophages and DCs stimulated via TLRs, but not in IL-10-producing T cells 7. In addition to NF-κB, other signaling cascades have been implicated in the regulation of IL-10 induction by TLR-activated macrophages and DCs, including the MAPKs ERK 6,8–12, and p38 8,9,13–17. ERK activation is required for IL-10 expression in different cells, from macrophages and DCs to Th cells 2. ERK activation upregulates the transcription factor cFOS, which in turn enhances IL-10 transcription 10,12 and increases the Il10 locus accessibility to the binding of transcription factors 8. Regulation of IL-10 transcription by p38 involves the transcription factor Sp1 13, the activation of the downstream kinases mitogen- and stress-activated protein kinase-1 and mitogen- and stress-activated protein kinase-2 and CREB phosphorylation 18 and the post-transcriptional regulation of IL-10 by macrophages in response to TLR4 ligation by helping to protect the *Il10* mRNA from rapid degradation induced by the RNA-binding protein tristetraprolin (TTP) 19. TTP deficient (^−/−^) macrophages show elevated levels of *Il10* mRNA upon TLR4 stimulation 20. Other post-transcriptional mechanisms for *Il10* gene regulation include the participation of certain microRNAs 21,22. Targeting mRNA stability is therefore an important mechanism for the regulation of IL-10 production. However, this mechanism has been mainly studied downstream of TLR4 and it is not clear how it operates downstream of other TLRs.

In this study, we compared the induction of IL-10 in macrophages stimulated via the TLR2 and TLR4 ligands, Pam3CysSerLys4 and LPS, respectively, as well as whole bacteria that predominantly signal through either of these TLRs. Independent of the stimuli, a peak of *Il10* mRNA was observed as early as 1 h post stimulation. However, whereas TLR2 signaling led to a rapid degradation of *Il10* mRNA, TLR4 signals contributed to increased stability of *Il10* mRNA, which was dependent on TRIF-mediated activation of the MAPK p38-signaling pathway. We thus provide evidence that the TRIF pathway regulates IL-10 production at the post-transcriptional level, discriminating between TLR2 and TLR4 activation of macrophages. Triggering of macrophages by TLR2- or TLR4-activating bacteria impacts IL-10 secretion by these cells, suggesting a potential relevance of the described mechanism for modulating the course of the immune response during infection.

## Results

### Distinct post-transcriptional regulation of IL-10 through TLR2 versus TLR4 signaling in macrophages

**Figure 1:**
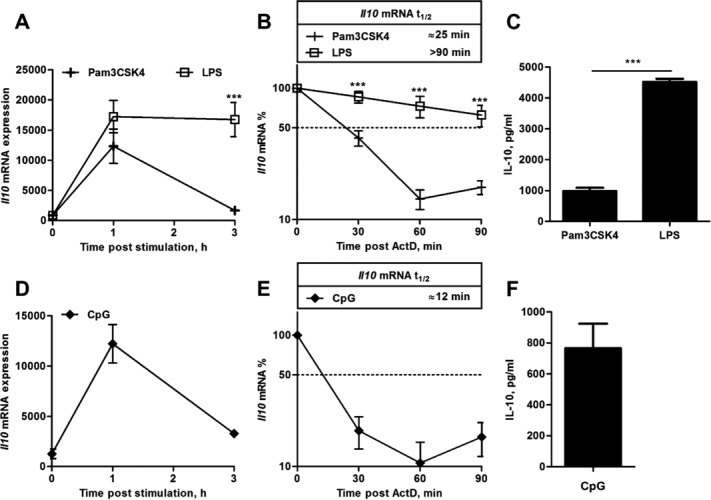
Distinct post-transcriptional regulation of IL-10 through TLR2 versus TLR4 signaling in BMMs. WT BMMs were stimulated with (A–C) Pam3CSK4 (2 μg/mL) or LPS (25 ng/mL) or with (D–F) CpG (1 μM). (A) At the indicated time-points post stimulation with Pam3CSK4 (crosses) or LPS (open squares) total RNA was harvested, converted to cDNA, and the *Il10* mRNA expression determined by real-time PCR and normalized to ubiquitin. (B) At 1 h post stimulation with Pam3CSK4 (crosses) or LPS (open squares), ActD was added to the cultures and 30, 60, or 90 min later the *Il10* mRNA expression determined as in (A). The percentage of *Il10* mRNA present at each time-point is relative to the amount of *Il10* mRNA measured at 1 h post stimulation in the absence of ActD. The *Il10* mRNA t_1/2_ was calculated as the time post ActD addition where 50% of *Il10* mRNA was still present. (C) Six hours post stimulation of BMMs with Pam3CSK4 or LPS, cell culture supernatants were collected and the amount of IL-10 measured by ELISA. (D–F) BMMs were stimulated with CpG (diamonds) and treated as in (A), (B), and (C), respectively. Data are shown as mean ± SEM of triplicates pooled from each of three (A–C) or two (D–F) independent experiments. ***p < 0.001; two-way ANOVA with a Bonferroni post test (A, B) or Student's *t*-test (C).

To dissect the molecular mechanisms regulating the initial steps of *Il10* gene expression in BM-derived macrophages (BMMs) stimulated via TLR2 or TLR4, we compared the kinetics of mRNA expression in response to ligands Pam3CSK4 and LPS. The dose of TLR2 and TLR4 agonists used corresponded to maximum IL-10 production by stimulated BMMs, as measured by immunoassay (Supporting Information Fig.1). Both stimuli induced a peak of *Il10* mRNA at 1 h post stimulation (Fig.1A). Strikingly, the *Il10* mRNA induced upon TLR2 activation of BMMs rapidly declined, with much lower levels detected at 3 h post stimulation (Fig.1A). This was in contrast to TLR4 triggering of BMMs, where the amount of *Il10* mRNA remained constant between 1 and 3 h post stimulation (Fig.1A). Since the profile of *Il10* mRNA observed upon TLR2 stimulation (Fig.1A) is compatible with a rapid degradation of *Il10* mRNA, the stability of the TLR2- or TLR4-induced *Il10* mRNA was assessed, by adding ActinomycinD (ActD) to the BMMs cultures at 1 h post stimulation. While the *Il10* mRNA induced by the TLR2 agonist was rapidly degraded upon the addition of ActD, *Il10* mRNA induced by TLR4 showed a prolonged t_1/2_ (Fig.1B). The IL-10 protein resulting of TLR2 versus TLR4 stimulation of BMMs was different, with higher quantities of IL-10 being secreted upon TLR4 activation (Fig.1C). At 3 h post stimulation with LPS the *Il10* mRNA was unstable (Supporting Information Fig.2A), suggesting that post-transcriptional mechanisms operate with either TLR, albeit with a different kinetics. We next investigated the *Il10* post-transcriptional regulation using another MyD88-dependent TLR stimulus. The *Il10* transcription and mRNA stability profile induced in BMMs upon TLR9 triggering with CpG, which like TLR2 signals via MyD88 alone, was similar to that induced upon TLR2 activation (Fig.1D, E, and F).

### TRIF signaling enhances the stability of *Il10* mRNA via prolonged p38 activation

**Figure 2:**
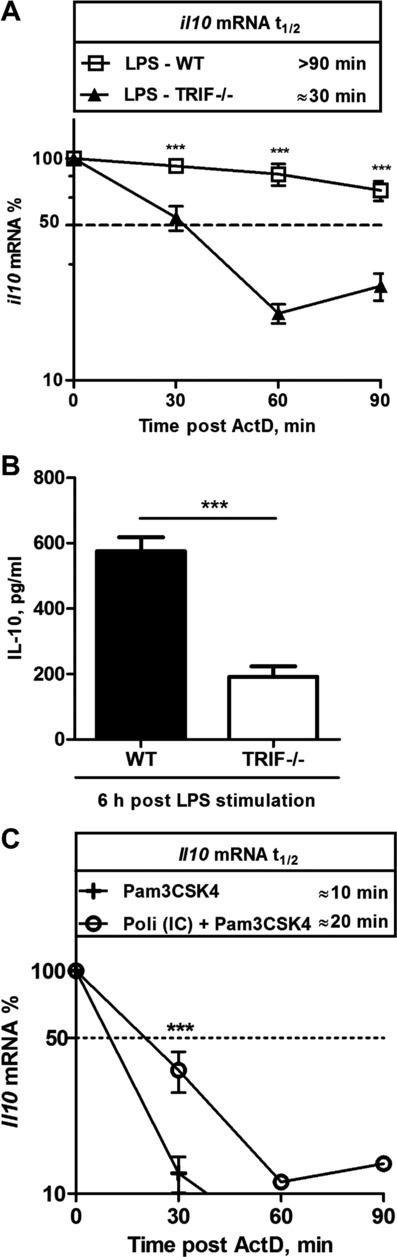
TRIF signaling enhances the stability of *Il10* mRNA. (A) BMMs generated from WT (solid line) or TRIF^−/−^ (dashed line) mice were stimulated with LPS (25 ng/mL) and the *Il10* mRNA t_1/2_ was determined at 1 h post stimulation, as described in Fig.1B. (B) Six hours post stimulation of WT (black bar) or TRIF^−/−^ (white bar) BMMs with LPS the cell culture supernatants were collected and the amount of IL-10 measured by ELISA. (C) WT BMMs were stimulated with Pam3CSK4 (2 μg/mL; crosses) alone or in combination with polyI:C (20 μg/mL; circles) and the *Il10* mRNA t_1/2_ was determined as before. Data are shown as mean + or ± SEM of triplicates pooled from each of two independent experiments. ***p < 0.001; two-way ANOVA with a Bonferroni post test (A, C) or Student's *t*-test (B).

Next, we investigated the molecular mechanism underlying the enhanced *Il10* mRNA stability upon TLR4 signaling. Since a major difference between TLR2 (and TLR9) versus TLR4-signaling pathways is the recruitment of TRIF in the case of TLR4 but not TLR2 (or TLR9), and as TRIF-dependent signals have been implicated in IL-10 production by macrophages 4, we hypothesized that TRIF may play a role in the observed differences. Therefore, BMMs from WT or TRIF^−/−^ mice were generated and stimulated with LPS for 1 h. At this time-point, ActD was added and the amount of *Il10* mRNA measured over time by real-time PCR. The absence of TRIF significantly decreased the stability of *Il10* mRNA upon TLR4 stimulation, leading to its degradation (Fig.2A). The amount of IL-10 protein detected in TLR4-stimulated cultures of TRIF^−/−^ BMMs was significantly lower than that observed for WT BMMs (Fig.2B). In the absence of TRIF, *Il10* transcription following TLR4 stimulation of BMMs was decreased (Supporting Information Fig.2B), suggesting that the TRIF pathway is also important to provide transcriptional enhancing signals. The *Il10* mRNA stability in BMMs stimulated with TLR2 alone or in combination with TLR3, where we expect to be triggering both MyD88 (via TLR2) and TRIF (via TLR3), was compared. The TLR2/TLR3 combined stimulation of BMMs led to a significant increase of the *Il10* mRNA half-life, in further support of a role for TRIF in stabilizing the *Il10* mRNA (Fig.2C). Stimulation of macrophages with the TLR3 agonist yielded very low amounts of *Il10* transcription in the first 3 h post stimulation (data not shown), making it impossible to analyze the stability of the mRNA in the context of single TLR3 stimulation.

**Figure 3:**
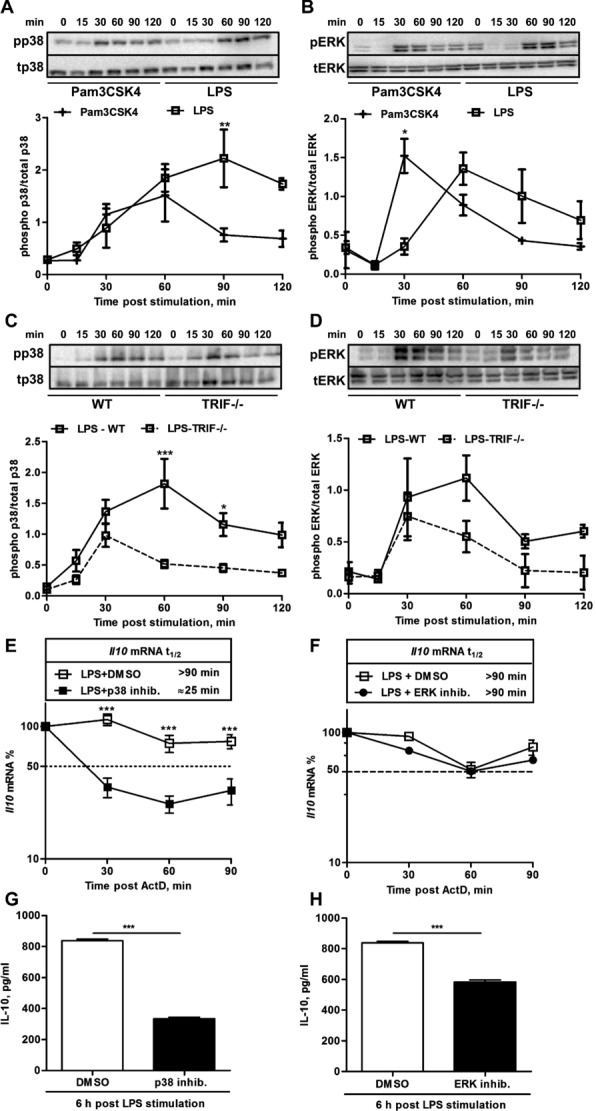
p38 mediates the TRIF-induced stability of *Il10* mRNA. (A, B) WT BMMs were stimulated with Pam3CSK4 (2 μg/mL; crosses) or LPS (25 ng/mL; open squares) and, at the indicated time points post stimulation, total cell extracts were prepared, separated in SDS-PAGE, and the ratio of (A) phospho p38/total p38 or (B) phospho ERK/total ERK was assessed by WB. (C, D) WT (solid line) or TRIF^−/−^ (dashed line) BMMs were stimulated with LPS (25 ng/mL) and the ratio of (C) phospho p38/total p38 or (D) phospho ERK/total ERK was assessed by WB as indicated in (A) and (B). WBs shown are from a single experiment with pooled protein extracts from three replicates per condition per time-point, representative of three independent experiments performed. Quantitative data are shown as mean ± SEM of results pooled from three independent experiments. (E, F) WT BMMs were stimulated with LPS for 50 min. At this time point, DMSO (as a control, open squares), (E) SB203580 (p38 inhibitor, at 2.5 μM; close squares) or (F) PD0325901 (ERK inhibitor, at 0.1 μM; close circles) were added to the cultures. Ten min later, ActD was added to all wells and the t_1/2_ of the *Il10* mRNA determined as in Fig.1B. (G, H) Cells were treated as before, but with no addition of ActD, and at 6 h post stimulation the supernatants were collected from (G) p38 inhibitor-treated and (H) ERK inhibitor-treated cells and the amount of IL-10 measured by ELISA. Data are shown as mean ± SEM of triplicates pooled from each of two independent experiments. *p < 0.05; **p < 0.01; ***p < 0.001; two-way ANOVA with a Bonferroni post test (A–F) or Student's *t*-test (G and H).

Both p38 and ERK have been extensively implicated in the regulation of *Il10* expression 2 and a role for p38 in the post-transcriptional regulation of *Il10* has also been described 19. We therefore investigated whether the differential activation of these MAPKs was involved in the observed post-transcriptional regulation of *Il10* via TLR4/TRIF. We compared the levels of p38 and ERK phosphorylation upon TLR2 and TLR4 stimulation of WT BMMs. Although over the first 60 min post stimulation the activation of p38 was similar between TLR2 and TLR4, after that time point, a decrease in p38 activation was observed in Pam3CSK4-stimulated cells, which was less pronounced in the case of LPS-stimulated cells (Fig.3A). TLR2 triggering led to a faster activation of ERK than TLR4 signaling, but the deactivation of this MAPK was similar to both signals (Fig.3B). We next investigated in more detail the enhanced p38 activation and found that it was dependent on TRIF signals (Fig.3C). ERK phosphorylation in the absence of TRIF was also decreased (Fig.3D). We then stimulated WT BMMs with LPS and, at 50 min post stimulation, added specific inhibitors for p38 or ERK. We chose to add the chemical inhibitors at 50 min post stimulation to minimize effects on the initial induction of *Il10* transcription. Inhibition of p38 led to the degradation of the *Il10* mRNA, whereas inhibition of ERK did not (Fig.3E and F). In line with this, inhibition of p38 activation had a stronger effect on IL-10 protein than ERK blockade (Fig.3G and H). Other p38 (BIRB) and ERK (PD184352 and U0126) inhibitors were used with similar results (data not shown). In summary, our data show that TRIF signals contribute to regulate IL-10 at the post-transcriptional level through a mechanism that involves enhanced p38 activation.

### TTP deficiency impacts *Il10* mRNA stability early upon TLR2 stimulation of BMMs

**Figure 4:**
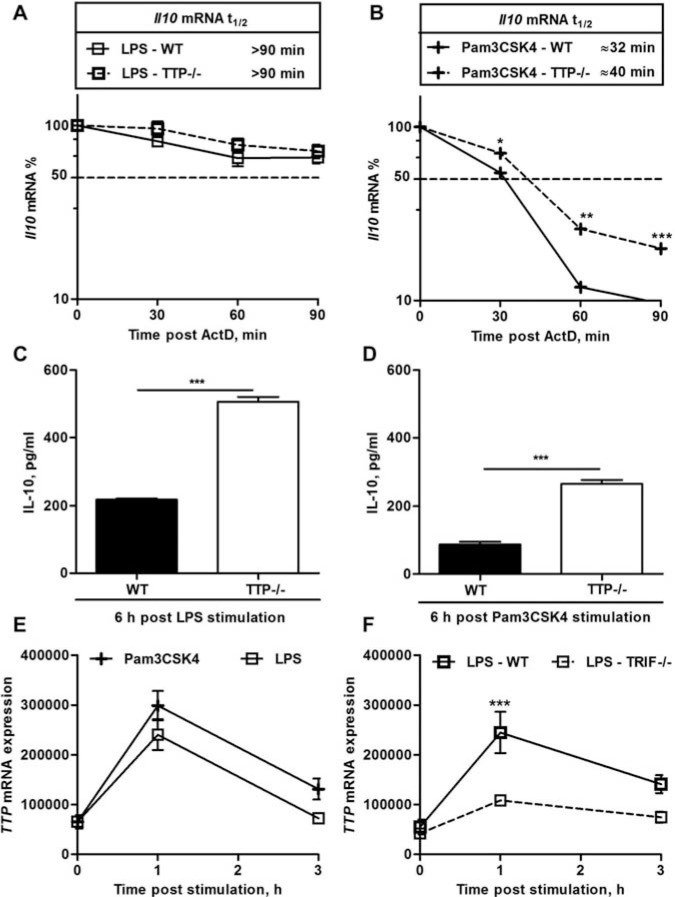
Absence of TTP delays the early degradation of the *Il10* mRNA induced upon TLR2-signaling. (A, B) WT (solid lines) or TTP^−/−^ (dashed lines) BMMs (generated from frozen cells) were stimulated with (A) LPS (25 ng/mL, open squares) or (B) Pam3CSK4 (2 μg/mL, crosses) for 1 h and the *Il10* mRNA t_1/2_ was determined upon addition of ActD as described in Fig.1B. (C, D) WT (black bars) or TTP^−/−^ (white bars) BMMs were generated and stimulated as above, except that ActD was not added, and the amount of IL-10 in the culture supernatants of (C) LPS-stimulated or (D) Pam3CSK4-stimulated cells was measured by ELISA 6 h post stimulation. (E) WT BMMs were stimulated with LPS (25 ng/mL, open squares) or Pam3CSK4 (2 μg/mL, crosses) and the TTP mRNA measured over time as indicated in Fig.1A. (F) WT (solid lines) or TRIF^−/−^ (dashed lines) BMMs were stimulated with LPS and the TTP mRNA measured over time as indicated in Fig.1A. All data are shown as mean + or ± SEM of triplicates pooled from each of two (A–D) or three (E, F) independent experiments. *p < 0.05; **p < 0.01; ***p < 0.001; the two-way ANOVA with a Bonferroni post test (A, B, E, and F) or Student's *t*-test (C, D).

The RNA-binding protein TTP targets *Il10* mRNA inducing its rapid degradation 19,20. p38 activation has been shown to inhibit TTP, thus promoting an increase of the t_1/2_ of *Il10* mRNA 19,20,23,24. Considering these reports and our findings showing that in TLR2-stimulated BMMs a rapid degradation of the *Il10* mRNA occurs, in parallel with a reduced activation of p38, we next investigated the stability of TLR2-induced *Il10* mRNA in the absence of TTP. WT or TTP^−/−^ BMMs were stimulated for 1 h with LPS or Pam3CSK4 and at that time ActD was added to the cultures and *Il10* mRNA measured by real-time PCR. LPS stimulation in the absence of TTP did not result in increased *Il10* mRNA stability at this time-point (Fig.4A). However, when the cells were stimulated with Pam3CSK4 a slight but significant increase in *Il10* mRNA t_1/2_ was observed in the absence of TTP (Fig.4B). For both LPS and Pam3CSK4 stimuli, an increase in IL-10 protein secretion was observed in TTP^−/−^ cells at 6 h post stimulation (Fig.4C and D). This is in line with previous reports showing that at later time points post stimulation with LPS, the absence of TTP increases the stability of the *Il10* mRNA with consequent increase on IL-10 production 19,20,23,24. We, herein, show that in the case of TLR2 activation, the absence of TTP influences *Il10* mRNA stability at an earlier time point than with TLR4. The transcriptional profile of *Ttp* observed in macrophages upon TLR2 or TLR4 activation was similar (Fig.4E) and the absence of TRIF decreased the expression of *Ttp* (Fig.4F). These data suggest that the differences observed for the stability of the *Il10* mRNA do not reflect or correlate to specific changes in the expression of *Ttp*.

### Differential regulation of IL-10 by TLR2 versus TLR4 upon bacterial stimulation of BMMs

**Figure 5:**
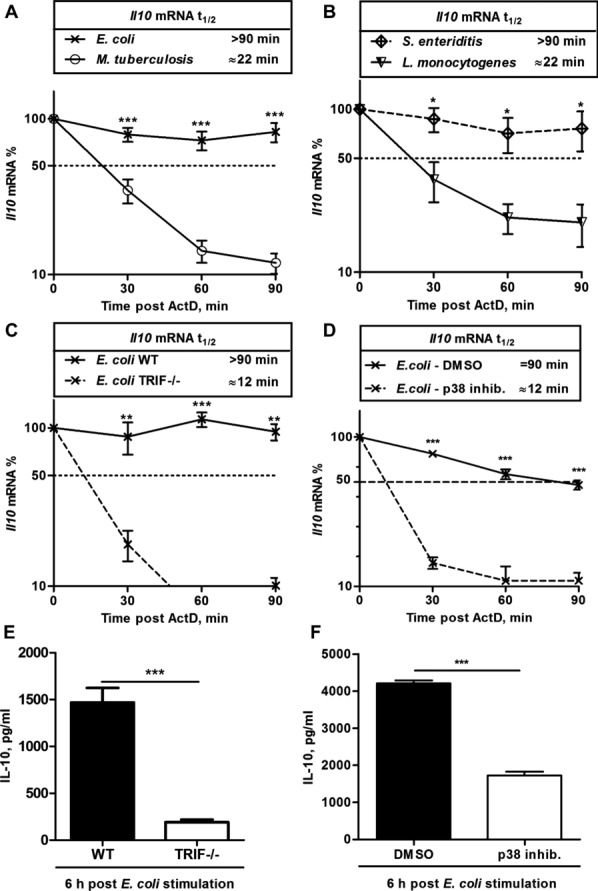
Differential post-transcriptional regulation of IL-10 by TLR2 versus TLR4 upon BMM stimulation with intact bacteria. (A, B) WT BMMs were stimulated with heat-killed (A) *Escherichia coli* (crosses), (B) *Salmonella enteritidis* (diamonds), (B) *Listeria monocytogenes* (inverted triangles) or with (A) live *Mycobacterium tuberculosis* H37Rv (open circles) at a MOI of 2. The *Il10* mRNA t_1/2_ was determined at 1 h post stimulation, as indicated in Fig.1B. (C) WT (solid lines) or TRIF^−/−^ (dashed lines) BMMs were stimulated with heat-killed *E. coli* (MOI of 2) and the *Il10* mRNA t_1/2_ determined at 1 h post stimulation, as indicated in Fig.1B. (D) WT BMMs were stimulated with heat-killed *E. coli* (MOI of 2) in the presence of DMSO (as a control, solid lines) or of SB203580 (p38 inhibitor, at 2.5 μM; dashed lines) and the *Il10* mRNA t_1/2_ determined at 1 h post stimulation, as indicated in Fig.1B. (E) Six hours post stimulation of WT (black bar) or TRIF^−/−^ (white bar) BMMs with *E. coli* the cell culture supernatants were collected and the amount of IL-10 measured by ELISA. (F) WT BMMs were stimulated with *E. coli* in the presence of DMSO (as a control, black bar) or of SB203580 (p38 inhibitor, white bar) for 6 h and the IL-10 protein present in the supernatants of the stimulated cultures determined by ELISA. All data are shown as mean + or ± SEM of triplicates pooled from each of three (A and B) or two (C–F) independent experiments. *p < 0.05; **p < 0.01; ***p < 0.001; two-way ANOVA with a Bonferroni post test (A–D) or Student's *t*-test (E, F).

Our findings unveil a novel link between TRIF and IL-10 post-transcriptional regulation, mediated by p38 and partly by TTP. However, these findings were obtained using TLR stimulation with chemically pure, single ligands. We sought to investigate if the described mechanism was also in place when BMMs were stimulated with bacteria. WT BMMs were stimulated with bacteria described in the literature to mainly require TLR2 ligation, such as *Mycobacterium tuberculosis* strain H37Rv 25 and *Listeria monocytogenes* 26, or TLR4 ligation, such as *Escherichia coli* and *Salmonella enteriditis* 27, for maximal BMMs activation. In agreement with the data from the respective TLR ligands, *M. tuberculosis* stimulation of BMMs, which requires TLR2 to induce IL-10, led to a rapid degradation of *Il10* mRNA, whereas *Il10* mRNA induced upon stimulation of these cells with *E. coli*, which predominantly induces IL-10 via TLR4, was stable over time (Fig.5A). *L. monocytogenes*, which signals mainly through TLR2 showed rapid degradation of induced *Il10* mRNA, whereas *S. enteriditis*, which mainly signals through TLR4 resulted in the induction *Il10* mRNA that was stable over time (Fig.5B).

To investigate whether TRIF and p38 activation also accounted for the increased stability of the *Il10* mRNA observed in response to bacteria signaling through TLR4, we further dissected the stimulation of BMMs with *E. coli*. Again *Il10* mRNA induced by *E. coli* stimulation of BMMs lost its stability in the absence of TRIF or p38 activation (Fig.5C and D). In addition, the amount of IL-10 protein secreted in the absence of TRIF or in the presence of the p38 inhibitor was lower than that obtained for WT cells in response to *E. coli* (Fig.5E and F). Additionally, the comparison of BMMs activation by *M. tuberculosis* H37Rv or *E. coli* showed that the relative amounts of *Il10* and *TTP* mRNA and of p38 activation followed the pattern described for TLR2 versus TLR4 activation with chemical agonists (Supporting Information Fig.3A–C). Also, in the absence of TRIF, the *Il10* and *TTP* mRNA and p38 activation were decreased in BMMs stimulated with *E. coli*, recapitulating the findings with LPS stimulation of these cells (Supporting Information Fig.3D–F).

Taken together, our study suggests that recognition of pathogens by distinct TLRs has an impact on the amount of IL-10 produced by BMMs. Our data demonstrate that this results from post-transcriptional mechanisms of *Il10* mRNA stabilization involving TLR4/TRIF/p38 signaling when BMMs are stimulated with TLR4-activating microbes. This reveals an important mechanism in modulating the course of the immune response.

## Discussion

IL-10 plays a fundamental role in regulating inflammation and the level of activation of adaptive immune responses 3 The regulation of *Il10* gene transcription induced by TLR activation has been an active area of research owing to the important immunoregulatory roles of this cytokine 2. Several studies demonstrate the existence of various layers for modulating IL-10 production, from epigenetic control, to transcriptional and post-transcriptional regulation 2.

We studied the molecular mechanisms leading to the post-transcriptional regulation of *Il10* mRNA induced by TLR2- versus TLR4-stimulated BMMs and found that TLR4 signals increase the t_1/2_ of the *Il10* mRNA, via enhanced p38 activation, which was dependent on TRIF. Despite initial transcriptional induction of the *Il10* gene at 1 h post stimulation, both TLR2 and TLR9 that lack the activation of the TRIF-signaling cascade, failed to sustain the t_1/2_ of *Il10* mRNA subsequent to this. Furthermore, providing TRIF signals together with TLR2 stimulation of BMMs led to an increase of the t_1/2_ of *Il10* mRNA. Our study confirms the role of TRIF for maximal IL-10 production by TLR4-stimulated BMMs 4, by promoting both a stronger transcription of the *Il10* gene and an increased stability of the *Il10* mRNA. Therefore, our study suggests that different TLRs regulate IL-10 expression in different ways, perhaps allowing the fine-tuning of IL-10 production to suit infections with different pathogens and/or commensals. Further studies are required to address whether the role of TRIF in enhancing p38 activity and *Il10* mRNA stability is direct or indirect. A possible candidate for an indirect role of TRIF in this process is type I IFN, since TLR2 agonists are poor inducers of IFN-β, as opposed to TLR4 ligands that induce IFN-β mRNA independently of MyD88 signaling 28. Also, IFN-β has been implicated in the induction and sustained expression of IL-10 by LPS-stimulated BMMs 28–31.

The mechanism proposed in our study was recapitulated upon stimulation of BMMs with microbes, such as *M. tuberculosis*, *L. monocytogenes*, *E. coli*, and *S. enteriditis*. Therefore, it is possible that the manipulation of specific TLR activation by bacteria will have implications on the amounts of IL-10 secreted by macrophages. In this sense, bacteria that preferentially trigger TLR4 may be manipulating the immune system to increase the amounts of IL-10, thus compromising the full efficacy of the immune response. It will be interesting to investigate if these differential pathways regulating IL-10 at the post-transcriptional level also occur in cells other than macrophages, or in macrophages located at different anatomical sites.

Studies of the 3′ untranslated regions of the *Il10* mRNA showed the existence of adenylate-uridylate rich elements (AREs), capable of mediating mRNA decay 32, providing evidence for post-transcriptional regulation of *Il10* mRNA. These ARE sequences recruit several ARE-binding proteins, such as TTP that was found to promote rapid mRNA decay of several transcripts, including that of TNF 33 and IL-10 19,20. In line with this, the t_1/2_ of *Il10* mRNA induced upon 5 h of LPS stimulation of macrophages was increased in the absence of TTP 20. As we now show, TTP also influences the t_1/2_ of the *Il10* mRNA induced by TLR2 activation, but in this case it targets the *Il10* mRNA earlier than observed for LPS. This difference between TLR2 and TLR4 is most likely related to differential TTP activation/inactivation, as the transcriptional pattern of TTP in BMMs activated through TLR2 or TLR4 is similar. Furthermore, TRIF deficiency led to less TTP transcription in LPS-stimulated BMMs than in WT cells. Altogether, our data suggest that a direct relation between TTP transcription and function is not in place, in support of the importance of TTP regulation by post-translational modifications. Specifically, the activity of TTP is negatively regulated by the MAPK p38 19,20,23,24. We observed a TRIF-mediated increase of p38 signaling, which is in line with previous studies 34,35, and associated it with an increased t_1/2_ of *Il10* mRNA. It is thus likely that the activation of TRIF and p38 upon TLR4 triggering are stabilizing the *Il10* mRNA in part through TTP inactivation. Of note, our data may look in apparent disagreement with previous studies showing that IL-10 negatively regulates p38 36,37. However, we observed stronger p38 activation during an initial phase of TLR4 stimulation (up to 120 min). During this initial period, the amount of IL-10 protein secreted by TLR2- or TLR4-stimulated BMMs is similar (data not shown), so, at this stage, differences in p38 are likely not related to differential regulation by IL-10. Inhibition of the MAPK ERK did not regulate IL-10 expression post-transcriptionally, but reduced the amount of IL-10 secreted by TLR4-stimulated BMMs, which is in line with previous reports 2. Thus, it is possible that ERK plays a major role in transcriptionally regulating IL-10, with p38 additionally participating at the post-transcriptional level. This observation suggests that the signaling cascades downstream of ERK and p38 diverge, for example, in what concerns TTP regulation.

TRIF signals appear therefore to enhance the activation of the MAPK p38, so that TTP-mediated mRNA degradation is delayed. One possible mediator of this mechanism is the MAPK phosphatase DUSP1. DUSP-1 was shown to impair p38 activity 38–40, to impair TTP expression and production by inhibiting p38 activation 41, and to induce the reduction of *Il10* mRNA stability 40. Also, DUSP1^−/−^ mice challenged in vivo with LPS showed increased production of IL-10 42. Since in response to LPS stimulation, TRIF^−/−^ macrophages showed decreased levels of DUSP1 activation relatively to WT cells 17, it is possible that the differences observed in terms of p38 activation are not directly related to DUSP1. Further studies are however needed to unequivocally answer this question.

The mechanism proposed in this study is likely to affect pro-inflammatory cytokines in addition to IL-10. Activation of the TRIF pathway with subsequent enhanced p38 activation and an increase in the t_1/2_ of cytokine mRNAs, promotes a specific post-transcriptional control that may shift the immune response towards a more inflammatory type. In this scenario, ensuring that IL-10 is also enhanced might be of importance for the achievement of a balanced response. IL-10 has been implicated in inducing its own transcription via STAT3 activation in human monocyte derived macrophages 43, which would constitute an autocrine loop for IL-10 induction. IL-10 induces the destabilization of its own mRNA possibly via a secondary factor 44. More recently, it has been shown that in addition to being a target of TTP, IL-10 is itself an activator of TTP, by reducing late p38 activity 37,45. Thus, in situations when IL-10 is being produced, TTP is activated, ensuring a shutdown of pro-inflammatory cytokines mediated by IL-10 induced mRNA decay. It is also possible that this mechanism subsequently limits IL-10 translation, thus guaranteeing the appropriate balance of the immune response. For both TLR2 and TLR4 stimulation of BMMs, early IL-10 protein was detected in similar amounts or was higher for TLR4 signals, thus suggesting that in our system, the differences in the stability of the *Il10* mRNA observed are likely not due to distinct IL-10 autocrine signaling.

The broad array of regulatory mechanisms in place to modulate IL-10 expression might be a consequence of IL-10 induction by a variety of stimuli in many different cell types. Uncovering the extensive network underlying these mechanisms will be of interest to targeted modulation of IL-10 production. As we show here, this extensive network differs with the type of stimuli and involves many layers of regulation, including at the post-transcriptional level. Although the early induction of *Il10* transcription in BMMs by TLR2 and TLR4 is similar, the cell then fine-tunes the amount of IL-10 via several mechanisms. Our preliminary data suggest that the regulation of transcription differs between TLR2 and TLR4 stimulation of macrophages. As shown here, another mechanism that regulates IL-10 differentially in TLR2 versus TLR4 macrophages is the post-transcriptional regulation mediated by TRIF/p38/TTP. Therefore, both transcriptional and post-transcriptional events likely cooperate for a stronger IL-10 production in the case of TLR4 stimulation. This mechanism operates in BMMs sensing whole bacteria and allows for distinct IL-10 induction by TLR2- versus TLR4-activating microbes. As a consequence, TLR4 stimulation of BMMs leads to higher levels of IL-10 production than TLR2 activation, which may be beneficial to inhibit inflammatory pathologies or on the other hand manipulated to the advantage of the pathogen.

## Materials and methods

### Animals

C57BL/6 females of 8–12 weeks of age were ordered from Charles River (Barcelona, Spain). TLR2^−/−^ 46 and TLR4^−/−^ 47 animals were bred and maintained at ICVS. TRIF^−/−^ 48 mice were from MRC-NIMR. All mouse protocols followed the European Union Directive 86/609/EEC and were previously approved by the national authority Direcção Geral de Veterinária.

### Cell culture

Complete Dulbecco modified Eagle's minimal essential medium (cDMEM) was prepared by supplementing DMEM with 10% FBS, 1% sodium pyruvate, 1% 4-(2-hydroxyethyl)-1-piperazineethanesulfonic acid and 1% l-glutamine (all from GIBCO). BMMs were generated in cDMEM supplemented with 20% of L929-cell conditioned media (LCCM). On day 0, 4 × 10^6^ cells in 8 mL were plated per Petri dish (Sterilin) and kept at 37°C and 5% CO_2_. On day 4, with 10 mL of cDMEM-20% LCCM was added per plate and BMMs were recovered on day 7, counted and stimulated for different time points as appropriate. TTP^−/−^ and control (WT) cells were from littermate male mice of 6–16 weeks of age that had been backcrossed 28 generations into C57Bl/6NTac mice and have been described previously 49; cells were used to derive BMMs in 30% of LCCM.

### Bacteria

*M. tuberculosis* H37Rv Pasteur, a kind gift from P. J. Cardona (Barcelona), was grown in Proskauer Beck medium containing 0.05% Tween 80 to mid-log phase and frozen in Proskauer Beck medium and 30% glycerol at −80°C. The DH5X strain of *E. coli* (ICVS) and *S. enteriditis* (a gift from J. Azeredo, INL) were grown in Lysogeny broth, to mid-log phase and frozen in PBS 1X and 30% glycerol at −80°C. *L. moncytogenes* (ICVS) was grown in Antibiotic Medium 3. *E. coli* was heat inactivated for 60 min at 95°C, *S. enteriditis* for 30 min at 72°C and *L. monocytogenes* for 60 min at 62°C prior to cell stimulation. All bacteria were added to the cell cultures at a multiplicity of infection of two.

### Reagents

LPS (*S. Minnesota*, Sigma), Pam3CSK4, and polyI:C (InvivoGen) were used at 25 ng/mL, 2 μg/mL, and 20 μg/mL, respectively. ActD (Sigma) was used at 10 μg/mL and added to the cell cultures 1 h post stimulation. p38 (SB 203580) and ERK (PD0325901) inhibitors, kind gifts of Professor Sir P. Cohen, were used at 2.5 and 0.1 μM 50, respectively, and added to the cell cultures 50 min post stimulation. Cell culture grade DMSO (Sigma) was used as a vehicle control.

### ELISA

IL-10 quantification was performed by ELISA following the manufacturer's instructions (eBioscience).

### RNA extraction, cDNA, and quantitative real-time PCR

Total RNA from stimulated and nonstimulated cell cultures was extracted with TRIzol® 143 Reagent (Invitrogen) and converted to cDNA according to the manufacturer's instructions (Fermentas). *Il10* and *Ttp* gene expressions were assessed by real-time PCR using SYBR Green (Fermentas) and TaqMan MasterMix (Applied Byosistems) and normalized against ubiquitin or Hypoxanthine Phosphoribosyltransferase 1 (*Hprt1*) expression, respectively, as previously described 7.

### Western blot (WB)

BMMs were rested for 5 h in 1% FBS–cDMEM prior to stimulation. At the indicated time points post stimulation, cell culture supernatants were discarded and cells gently washed with apyrogenic 1X PBS (GIBCO). Protein extracts were obtained with a lysis buffer solution (100 mM Tris-HCl pH8; 10% glycerol; 1 mM EDTA pH8; 5 mM MgCl2; 50 mM NaCl; 1% NP-40; 1 × protease inhibitor cocktail (Roche); 1 × phosphatase inhibitor cocktails II and III (Sigma); dH2O) for 20 min on ice. Extracts were kept at −80°C until further use. Samples were then centrifuged at 13 000 rpm for 15 min and protein extracts recovered. Immediately before use, protein extracts were heated 5 min at 95°C and 20 μg of each sample resolved in a 12% SDS-PAGE and then transferred to nitrocellulose membranes. Total and phospho p38 (Threonine 180 and Tyrosine 182) and total and phospho ERK (Threonine 202/185 and Tyrosine 204/187) were detected by using specific antibodies (Cell Signaling). The membranes were developed with SuperSignal Femto substrate (Thermo Scientific) and read by a Universal Hood II (Bio-Rad). Quantity one (Bio-Rad) software was used to analyze the results.

### Statistical analysis

Data are expressed as mean + or ± SEM and analyzed by the two-way analysis-of-variance (ANOVA) test with a Bonferroni post test or by the two-tailed Student's *t*-test, as indicated. The *p* values considered as having statistical significance were *p ≤ 0.1; **p ≤ 0.01; and ***p ≤ 0.001.

## Abbreviations

ARE: adenylate-uridylate rich element
ANOVA: analysis-of-variance
BMM: BM-derived macrophage
cDMEM: complete Dulbecco modified Eagle's minimal essential medium
LCCM: L929-cell conditioned media
Pam3CSK4: Pam3CysSerLys4
TRIF: Toll/IL-1 receptor domain-containing adaptor inducing IFN-β
TTP: tristetraprolin
WB: Western Blot

## Supporting Information

As a service to our authors and readers, this journal provides supporting information supplied by the authors. Such materials are peer reviewed and may be re-organized for online delivery, but are not copy-edited or typeset. Technical support issues arising from supporting information (other than missing files) should be addressed to the authors.

**Figure S1**. IL-10 production by BMMs in response to increasing doses of Pam3CSK4 or LPS. **Figure S2**. (A) WT BMMs were stimulated with LPS (25 ng/mL) and 1h (open squares) or 3 h (inverted 6 triangles) later the *Il10* mRNA t1/2 determined as indicated in Figure 1B. **Figure S3**. (A, B and C) WT BMMs were stimulated with live *M. tuberculosis* H37Rv (open circles) or 12 with heat-killed *E. coli* (crosses) at a moi of 2.
