# Supplementary material for: Differential post-transcriptional regulation of IL-10 by TLR2 and TLR4-activated macrophages
Source: Eur J Immunol. 2013 Dec 16;44(3):856–66. doi: 10.1002/eji.201343734 (PMC4623319; doi:10.1002/eji.201343734)
Supplement: Supplementary file 1 — Figure S1. IL-10 production by BMMs in response to increasing doses of Pam3CSK4 or LPS. Figure S2. (A) WT BMMs were stimulated with LPS (25 ng/mL) and 1h (open squares) or 3 h (inverted 6 triangles) later the Il10 mRNA t1/2 determined as indicated in Figure 1B. Figure S3. (A, B and C) WT BMMs were stimulated with live M. tuberculosis H37Rv (open circles) or 12 with heat-killed E. coli (crosses) at a moi of 2. [file eji0044-0856-sd1.pdf]

European Journal of Immunology

Supporting Information

for

DOI 10.1002/eji.201343734

Maria Teixeira-Coelho, Joana Guedes, Pedro Ferreirinha, Ashleigh Howes, Jorge Pedrosa, Fernando Rodrigues, Wi S. Lai, Perry J. Blackshear, Anne O´Garra, António G. Castro and Margarida Saraiva

Differential post-transcriptional regulation of IL-10 by TLR2 and TLR4-activated macrophages

© 2013 The Authors. *European Journal of Immunology* published by WILEY-VCH Verlag GmbH & Co. KGaA Weinheim.

Supporting Information

Figure 1. IL-10 production by BMMs in response to increasing doses of Pam3CSK4 or LPS. WT BMMs were stimulated with the indicated doses of (A) Pam3CSK or (B) LPS and 6 h post-stimulation the amount of IL-10 in the culture supernatants measured by ELISA. Represented is the mean ± SEM of two independent experiments. Each time point represented was performed in triplicate for each experiment.

Figure 2. (A) WT BMMs were stimulated with LPS (25 ng/mL) and 1h (open squares) or 3 h (inverted triangles) later the *Il10* mRNA $t_{1/2}$ determined as indicated in Figure 1B. (B) WT (solid lines) or TRIF^{-}/$^{-}$ (dashed lines) BMMs were stimulated with LPS (25 ng/mL) and at the indicated time points the *Il10* mRNA determined as in Figure 1A. Represented is the Mean ± SEM of two independent experiments. Each time point represented was performed in triplicate for each experiment. *p* values were determined by the two-way ANOVA with a Bonferroni post-test; ***p<0.001.

Figure 3. (A, B and C) WT BMMs were stimulated with live *M. tuberculosis* H37Rv (open circles) or with heat-killed *E. coli* (crosses) at a moi of 2. At the indicated time points post-stimulation the expression of *Il10* (A) or *TTP* (B) mRNA was determined as detailed in Figure 1A. The ratio of phospho p38/total p38 (C) was also determined as detailed in Figure 3A. (D-F) WT (solid lines) or TRIF^{-}/$^{-}$ (dashed lines) BMMs were stimulated with heat-killed *E. coli* (moi of 2) and the expression of *Il10*, *TTP* or the ratio phospho p38/total p38 were determined as before. Represented is the mean ± SEM of two independent experiments. Each time point represented was performed in triplicate for each experiment. *p* values were determined by the two-way ANOVA with a Bonferroni post-test; * *p*<0.05; ** *p*<0.01; ****p*<0.001.

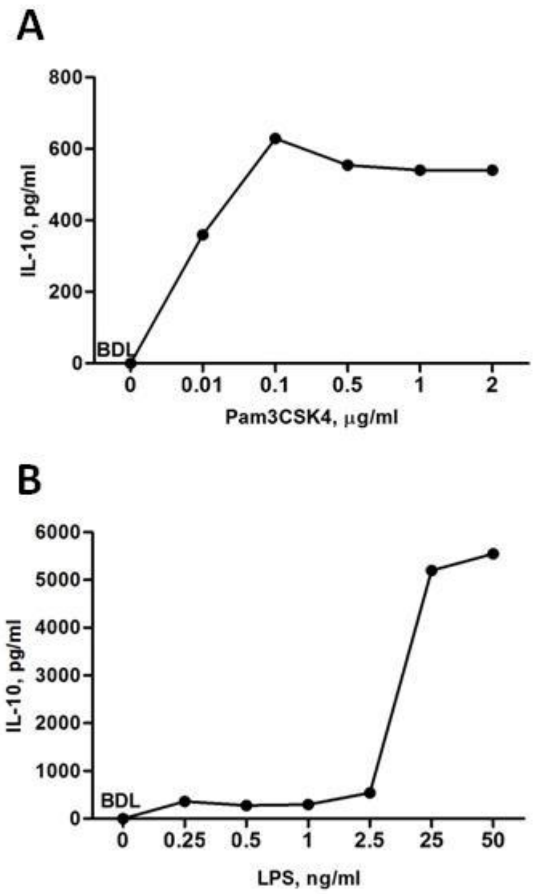

Supplementary Figure 1

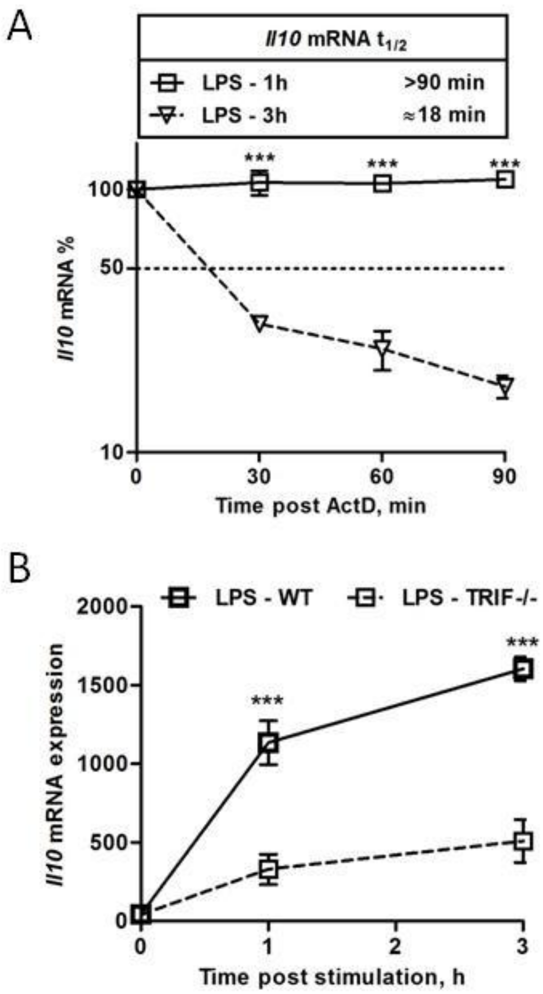

Supplementary Figure 2

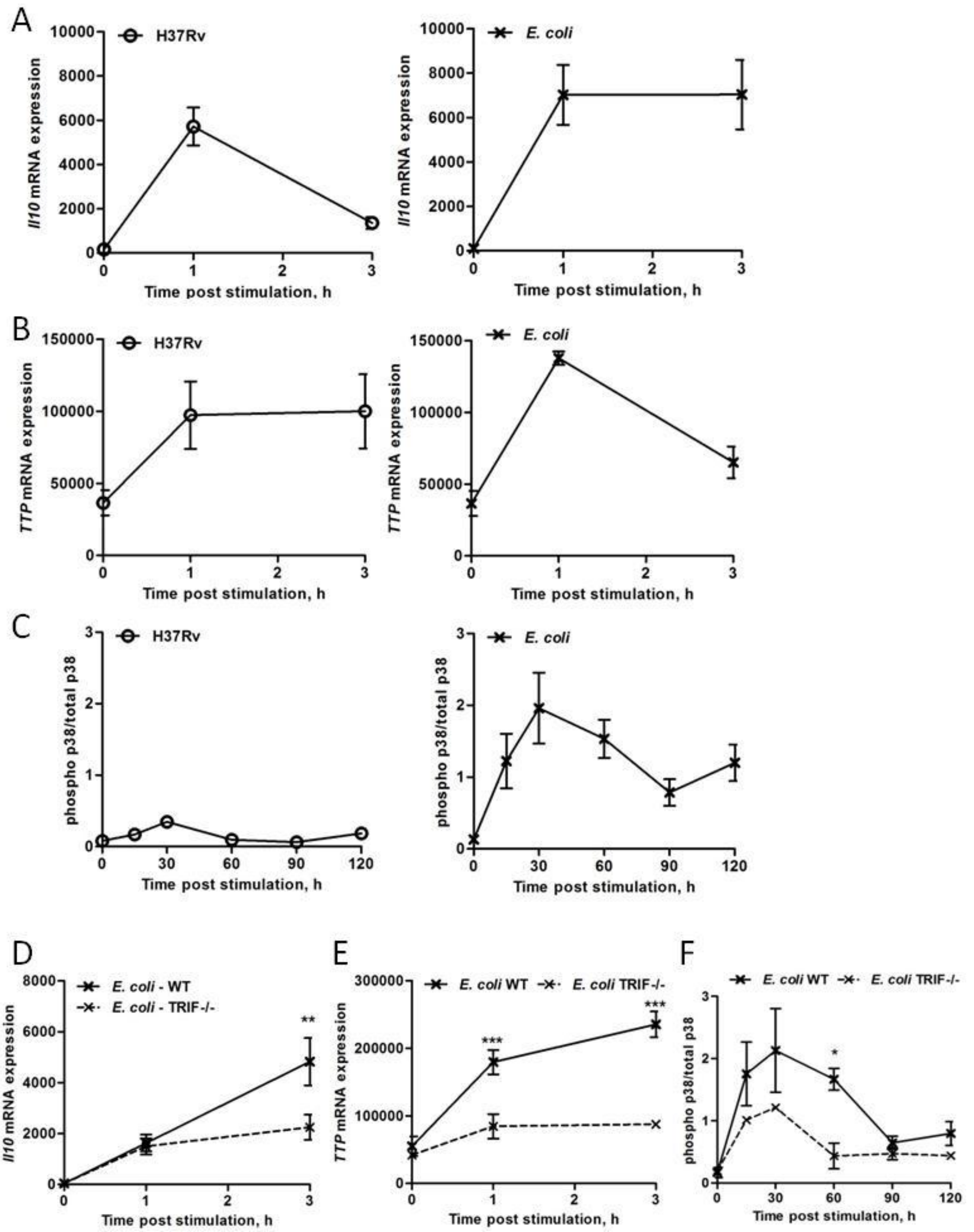

Supplementary Figure 3
